# Supplementary material for: Identification of photoperiod-induced specific miRNAs in the adrenal glands of Sunite sheep (Ovis aries)
Source: Front Vet Sci. 2022 Jul 22;9:888207. doi: 10.3389/fvets.2022.888207 (PMC9354845; doi:10.3389/fvets.2022.888207)
Supplement: Supplementary file 1 [file Data_Sheet_1.ZIP › Supplementary file/miRDeep2 file.zip!/miRDeep2 file/Know_Structure/SPLP42A2/oar-mir-374a.pdf]

miRBase precursor : oar-mir-374a
Total read count : 25902
oar-miR-374a read count : 21903
remaining reads : 3999

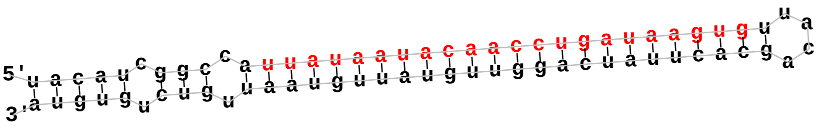

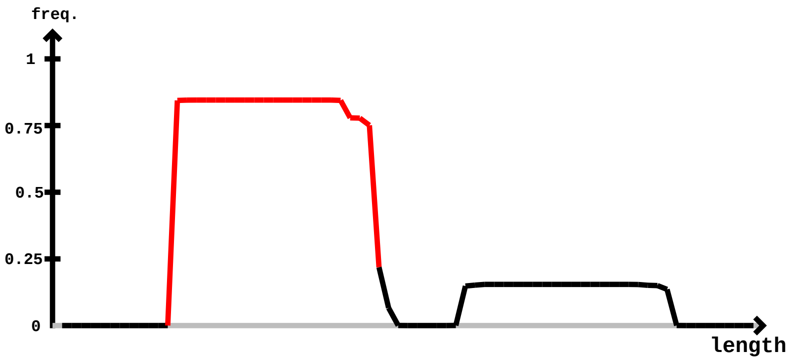


```
                oar-miR-374a
5'- uacaucggccauuauaauacaaccugauaaguguuacagcacuuaucagguuguauuguaauugucugugua  -3'   exp
    (((((.(((.((((((((((((((((((((((((....)))))))))))))))))))))))).))).)))))   reads   mm   sample
    ..........auuauaauacaaccugauaagu........................................   2       0    seq
    ...........uuauaauacaaccugaA............................................   1       1    seq
    ...........uuauaauacaaccugau............................................   33      0    seq
    ...........uuaCaauacaaccugau............................................   1       1    seq
    ...........uuauUauacaaccugaua...........................................   10      1    seq
    ...........uCauaauacaaccugaua...........................................   4       1    seq
    ...........Cuauaauacaaccugaua...........................................   14      1    seq
    ...........uuauaauacaaccugaua...........................................   1590    0    seq
    ...........uuaCaauacaaccugaua...........................................   22      1    seq
    ...........uuauaauacaaccugGua...........................................   5       1    seq
    ...........uuauaauacaaccugauU...........................................   25      1    seq
    ...........uuauaauacaacAugaua...........................................   1       1    seq
    ...........uuauaauacaUccugaua...........................................   7       1    seq
    ...........uuaAaauacaaccugaua...........................................   15      1    seq
    ...........uuauaauacaaccugauaa..........................................   26      0    seq
    ...........uuauaauacaaccugGuaag.........................................   3       1    seq
    ...........uuauaauacaaccugauaag.........................................   688     0    seq
    ...........uuaCaauacaaccugauaag.........................................   6       1    seq
    ...........uuauaauacaaccugauaagu........................................   13607   0    seq
    ...........uuaCaauacaaccugauaagu........................................   158     1    seq
    ...........uGauaauacaaccugauaagu........................................   2       1    seq
    ...........uuauaauacaaccugauaagug.......................................   3894    0    seq
    ...........uuaCaauacaaccugauaagug.......................................   47      1    seq
    ...........uuauaauacaaccugauaagugu......................................   1700    0    seq
    ...........uuauaauacaaccugauaaguguu.....................................   5       0    seq
    ............uauaauacaaccugaua...........................................   1       0    seq
    ............uauaauacaaccugauaagu........................................   17      0    seq
    ............uauaauacaaccugauaagug.......................................   5       0    seq
    ............uauaauacaaccugauaagugu......................................   8       0    seq
    ............uauaauacaaccugauaaguguu.....................................   1       0    seq
    .............auaauacaaccugauaagG........................................   1       1    seq
    .............auaauacaaccugauaagu........................................   3       0    seq
    .............auaauacaaccugauaagugu......................................   1       0    seq
    ...............aauacaaccugauaaguguuacagcacuuau..........................   1       0    seq
    ........................................acuuaucagguuguauuguaau..........   4       0    seq
```

oar-miR-374a

```
uacaucggccauuauaauacaaccugauaaguguuacagcacuuaucagguuguauuguaauugucugugua
........................................acuuaucagguuguauuguaauu.........    5       0       seq
.........................................cuuCucagguuguauugu.............    1       1       seq
.........................................cuuaucagguuguauugu.............    10      0       seq
.........................................cuuaucagguuAuauugua............    1       1       seq
.........................................cuuaucagguuguauuAua............    2       1       seq
.........................................cuuCucagguuguauugua............    7       1       seq
.........................................cuuaucagguuguauugua............    65      0       seq
.........................................cuuaucagguuguauuguaa...........    27      0       seq
.........................................cuuaucagguuguauuguaau..........    349     0       seq
.........................................cuuaucagguuAuauuguaau..........    1       1       seq
.........................................cuuaucagguuguauuguaauu.........    3357    0       seq
.........................................cuuaucagguuAuauuguaauu.........    3       1       seq
.........................................cuuaucagguuguauuguaauug........    6       0       seq
..........................................uuaucagguuguauuguaa...........    2       0       seq
..........................................uuaucagguuguauuguaau..........    12      0       seq
..........................................uuaucagguuguauuguaauu.........    74      0       seq
..........................................uuaucagguuguauuguaauug........    1       0       seq
...........................................uaucagguuguauuguaau..........    3       0       seq
...........................................uaucagguuguauuguaauu.........    65      0       seq
...........................................uaucagguuguauuguaauug........    1       0       seq
...........................................uaucagguuguauuguaauugu.......    1       0       seq
.............................................ucagguuguauuguaauu.........    1       0       seq
```
